# The cardioprotective effects and mechanisms of naringenin in myocardial ischemia based on network pharmacology and experiment verification

**DOI:** 10.3389/fphar.2022.954555

**Published:** 2022-09-09

**Authors:** Yakun Yang, Jiaying Qi, Muqing Zhang, Pingping Chen, Yanshuang Liu, Xiaorun Sun, Li Chu

**Affiliations:** ^1^ School of Pharmacy, Hebei University of Chinese Medicine, Shijiazhuang, Hebei, China; ^2^ College of Integrative Medicine, Hebei University of Chinese Medicine, Shijiazhuang, Hebei, China; ^3^ College of Basic Medicine, Hebei University of Chinese Medicine, Shijiazhuang, Hebei, China; ^4^ Hebei Key Laboratory of Integrative Medicine on Liver-Kidney Patterns, Institute of Integrative Medicine, College of Integrative Medicine, Hebei University of Chinese Medicine, Shijiazhuang, Hebei, China; ^5^ Hebei Key Laboratory of Chinese Medicine Research on Cardio-Cerebrovascular Disease, College of Integrative Medicine, Hebei University of Chinese Medicine, Shijiazhuang, Hebei, China

**Keywords:** naringenin, myocardial ischemia, network pharmacology, oxidative stress, apoptosis, l-type Ca 2+ currents

## Abstract

Naringenin (Nar) is a natural flavonoid extracted from citrus fruits with abundant pharmacological properties against cardiac diseases, but existing studies are unsystematic and scattered. The present research systematically investigates the mechanism of action of Nar in the treatment of myocardial ischemia (MI). Network pharmacology was used to analyze the relevant targets of Nar against MI as well as the biological mechanisms. The protective effect of Nar was initially assessed in H9c2 cells induced by CoCl_2_. In acutely isolated rat cardiomyocytes, Nar was further explored for effects on L-type Ca^2+^ currents, cell contractility and Ca^2+^ transients by using patch-clamp technique and Ion Optix system. Network pharmacology analysis indicated that Nar improved apoptosis, mitochondrial energy metabolism, inflammation and oxidative stress. Experimental validation demonstrated that Nar decreased ROS and MDA levels and increased antioxidant activity (e.g., GSH-P_X_, SOD, and CAT), mitochondrial membrane potential, ATP and Ca^2+^-ATPase contents. Nar also markedly reduced inflammatory factor levels, apoptosis, and intracellular Ca^2+^ concentrations in H9c2 cells. Based on the experimental results, it is speculated that Ca^2+^ signals play an essential role in the process of Nar against MI. Thus, we further confirmed that Nar significantly inhibited the L-type Ca^2+^ currents, contractility and Ca^2+^ transients in acutely isolated cardiomyocytes. The inhibition of Ca^2+^ overload by Nar may be a novel cardioprotective mechanism. The present study may serve as a basis for future clinical research, and Nar as a Ca^2+^ channel inhibitor may provide new perspectives for the treatment of myocardial ischemic diseases.

## Introduction

The World Health Organization has proposed a 25% reduction in premature cardiovascular disease mortality by 2025 (Roth et al., 2015; Joseph et al., 2017). Myocardial ischemia (MI) is a pathological state that prevents normal cardiac function, and is the most common cause of cardiovascular disease. The sudden decrease in blood supply to the heart leads to a reduced oxygen supply to the heart and abnormal cardiac energy metabolism. The underlying mechanisms of MI are closely related to mitochondrial dysfunction (Hausenloy & Yellon, 2013), oxidative stress (Hausenloy & Yellon, 2013; Shanmugam, Ravindran, Kurian, & Rajesh, 2018), and Ca^2+^ overload (Hausenloy & Yellon, 2013; H. Zhou, Wang, Zhu, Hu, & Ren, 2018).

Mitochondrial abnormalities have long been known to be a key pathological process leading to MI injury (Bugger & Pfeil, 2020). In such cases, the electron transport chain (ETC) does not work properly, thus resulting in ATP depletion; however, this depletion is not adequately compensated for by increased glycolysis (Lopaschuk, Ussher, Folmes, Jaswal, & Stanley, 2010). Acidic conditions prevent the opening of the mitochondrial permeability transition pore (MPTP) and excessive contraction of cardiomyocytes (Hausenloy & Yellon, 2013). High generation of reactive oxygen species (ROS) in the reperfusion process leads to decreased systolic and diastolic function and cell death in cardiomyocytes. ROS-mediated oxidative stress is the main pathological condition of myocardial damage (Chouchani et al., 2014). The production of large amounts of ROS leads to impaired mitochondrial function, which in turn reduces ATP content. The sarcoplasmic reticulum (SR) then cannot utilize excess Ca^2+^ from the cytosol due to a lack of energy leading to a large accumulation of calcium ions (Dewenter, von der Lieth, Katus, & Backs, 2017). During myocardial ischemia, myocardial cells lack oxygen and nutrient supply, which in turn leads to the development of cellular acidosis (Hausenloy & Yellon, 2013). Reverse activation of the sodium-calcium exchanger (NCX) leads to intracellular Ca^2+^ overload. In addition, ATP deficiency leads to a decline in Ca^2+^-ATPase activity at the plasma membrane and an increase in intracellular Ca^2+^ overload (Lu et al., 2020). Ca^2+^ overload is the final pathway for irreversible damage in the process of myocardial ischemia. Therefore, tools to mitigate MI damage are urgently needed.

Numerous epidemiological studies have demonstrated the safe and effective cardioprotective properties of flavonoids (Jiang, Chang, & Dusting, 2010). Naringenin (Nar) is a natural flavonoid extracted from the citrus genus with rich pharmacological characteristics. Nar exerts cardioprotective effects by activating mitochondrial large-conductance calcium-regulated potassium channels (mitoBKCachannel) (Testai et al., 2017; Kicinska et al., 2020). Nar alleviates myocardial ischemia-reperfusion (MI/R) injury by directly inhibiting mitochondrial oxidative stress damage and protecting mitochondrial biogenesis (L. M. Yu, Dong, Xue, et al., 2019). In addition, Nar can inhibit MI/R-induced endoplasmic reticulum stress and oxidative stress (Tang et al., 2017; L. M. Yu, Dong, Zhang, et al., 2019). However, the protective effects of Nar on myocardial ischemic damage and its mechanism are not completely understood.

**FIGURE 1:**
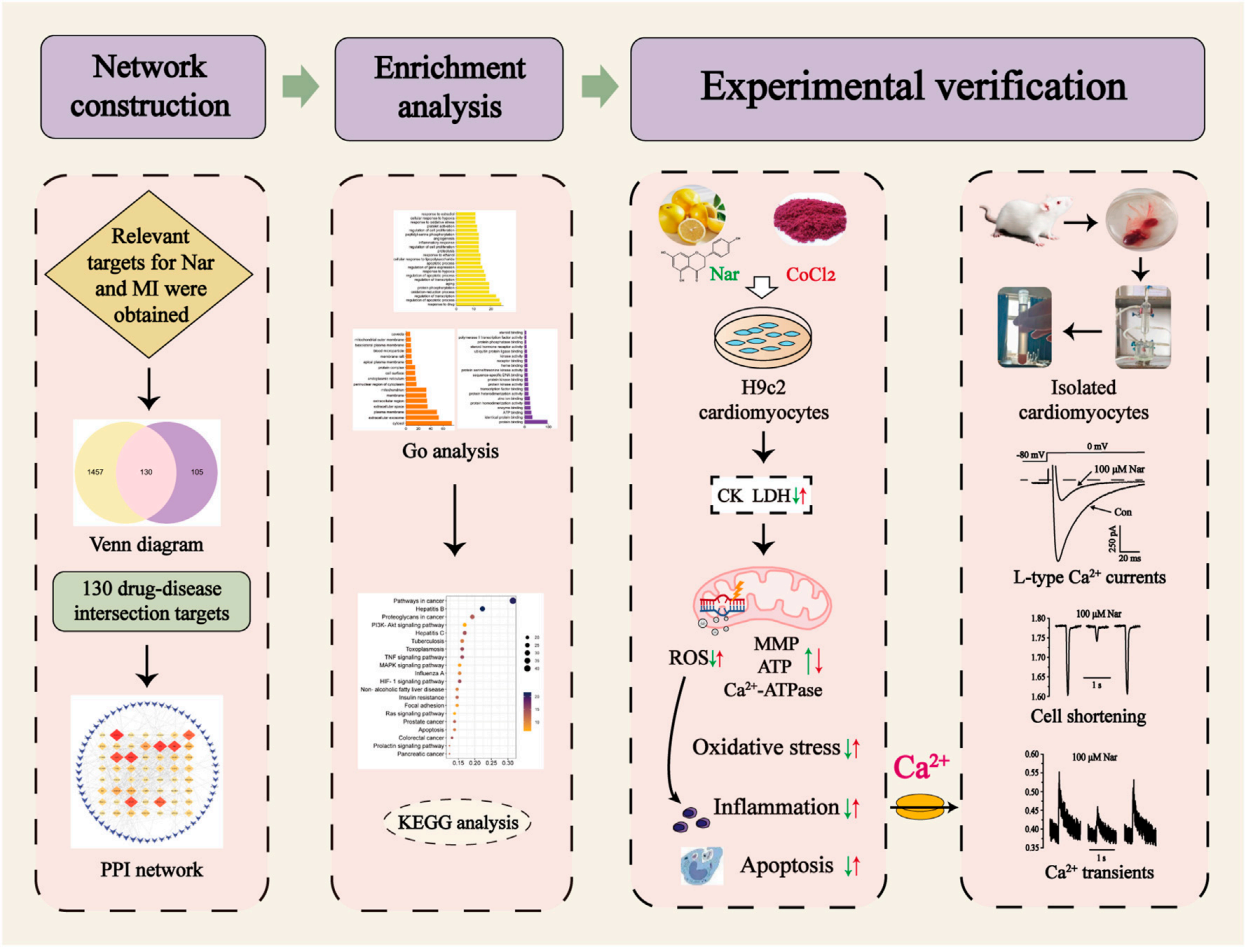
Graphical abstract of the study.

Network pharmacology has been used as a new research method to successfully reveal multitarget and complex mechanisms of drugs in various diseases. Systematic network analysis explains the underlying mechanism of Nar treatment of MI and provides the foundation for subsequent experimental validation. This study is to apply network pharmacology to predict the relevant targets for Nar treatment of MI. It was experimentally validated using CoCl_2_-induced H9c2 cardiomyocytes. In acutely isolated rat cardiomyocytes, Nar was further explored for effects on L-type Ca^2+^ current (I_Ca-L_), cell contractility and Ca^2+^ transients by using patch-clamp technique and Ion Optix system (Figure 1). The current research provides the biological information and experimental basis for further clinical applications of Nar.

## Materials and methods

### Network pharmacology prediction

#### Acquiring targets of naringenin and myocardial infarction

The 2D Structure and SMILES information of Nar were collected from PubChem (https://pubchem.ncbi.nlm.nih.gov/). The Traditional Chinese Medicine Systems Pharmacology Database (TCMSP, https://old.tcmsp-e.com/index.php), Comparative Toxicogenomic Database (CTD, https://ctdbase.org/), PharmMapper (http://lilab-ecust.cn/pharmmapper/) and Swiss TargetPrediction (http://swisstargetprediction.ch/) were used to search for the target corresponding to naringenin. The standard gene names of target proteins were gained from UniProtKB (https://www.uniprot.org/). Here, search for disease-related targets in three databases using the keyword "myocardial ischemia”, including the GeneCards database (https://www.genecards.org/), Database of Gene-Disease Associations (DisGeNET, https://www.disgenet.org/) and Online Mendelian Inheritance in Man (OMIM, https://www.omim.org/).

#### Construction of the PPI network and analysis

The targets associated with MI and Nar were entered into an online Venn analysis tool (https://bioinfogp.cnb.csic.es/tools/venny/) to create Venn diagrams and obtain drug-disease intersection targets. The protein-protein interaction (PPI) network of the intersection targets was then constructed in the string database (https://string-db.org/). The “string.tsv” file was downloaded, and Cytoscape 3.6.1 was used to create a network of MI-Nar intersection targets. Finally, the Database for Annotation, Visualization and Integrated Discovery (DAVID) database (https://david.ncifcrf.gov/) was used for GO and KEGG analysis to visualize and analyze the obtained data. In addition, *p* values <0.01 were set to better predict and validate biological processes and mechanisms.

### Chemical reagents

Nar (98% purity) was provided by Chengdu Alfa Biotechnology Co., Ltd. (Chengdu, China). Nar was dissolved in dimethyl sulfoxide (DMSO) to prepare the concentrations required for subsequent experiments. Penicillin/streptomycin and trypsin were purchased from Leagene Biotechnology (Beijing, China). Phosphate-buffered saline (PBS) was provided by Solarbio Life Sciences (Beijing, China). High-glucose Dulbecco’s Modified Eagle Medium (DMEM) and fetal bovine serum (FBS) were supplied by Gibco (Thermo Fisher Scientific, Waltham, USA). Cell Counting Kit-8 (CCK-8) was acquired from Beijing Zoman Bio-technology Co., Ltd. (Beijing, China). Collagenase type II was obtained from Worthington Biochemical (Lakewood, USA). Isoproterenol (ISO) and verapamil (Ver) were provided by Hefeng Pharmaceutical Co., Ltd. (Shanghai, China). All other reagents are of analytical quality and were obtained from Sigma Chemical Company (MO, USA).

All kits used here were obtained from Jiancheng Bioengineering Institute (Nanjing, China): CK (Catalog: A032-1-1), SOD (Catalog: A001-3-2), LDH (Catalog: A020-1-2), BCA (Catalog: A045-3), CAT (Catalog: A007-1-1), MDA (Catalog: A003-1-2), GSH-P_X_ (Catalog: A005-1-2), ATP (Catalog: A095-1-1), Ca^2+^-ATPase (Catalog: A016-1-2), TNF-α (Catalog: H007-1-1), and IL-6 (Catalog: H052-1).

### Cell culture and treatment

H9c2 cardiomyocytes, a subclonal cell line derived from embryonic rat myocardium, were provided by the Bluefbio (Shanghai, China). H9c2 cells were cultured in DMEM containing 100 U/ml penicillin/streptomycin and 10% FBS and maintained in a 5% CO_2_ incubator at 37°C. Cells were then subcultured or inoculated into 96-well plates according to experimental design.

Cobalt chloride (CoCl_2_) is used as a common chemical hypoxic agent to simulate hypoxic/ischemic conditions in a variety of cells (Pecoraro, Pinto, & Popolo, 2018; Han, Zhang, Dong, Wang, & Wang, 2021). Based on the results of the CCK-8 assay, Nar and CoCl_2_ concentrations were determined. Cells inoculated in 96-well plates were randomly divided into four groups: 1) control group (Con); 2) CoCl_2_; 3) L-Nar + CoCl_2_; and 4) H-Nar + CoCl_2_. Incubation with CoCl_2_ (600 μM) for 24 h was used to simulate an ischemic environment. H9c2 cells were pretreated with Nar (10 μM or 30 μM) for 4 h before CoCl_2_ treatment. Using the CCK-8 kit, the viability of the cells was determined after the experiment.

### Assessment of biochemical analysis

Cells were inoculated in 6-well plates after reaching 70–80% confluence. Drug treatment was consistent with the study above. The supernatant was collected for analysis after treatment. CK and LDH levels were measured via their corresponding kits. The manufacturer’s requirements were followed during all of the experimental steps.

H9c2 cells were digested with trypsin and collected, followed by centrifugation at 1,000 rpm/min for 10 min to collect the precipitated cells. PBS (pH 7.2-7.4) was added to the cell precipitate for suspension, and the cells were centrifuged again in similar conditions. Next, the supernatant was removed, and the cells were collected for ultrasonic disruption. BCA is used to determine the protein concentration in each. The SOD, CAT, GSH-P_X_, MDA, ATP, Ca^2+^-ATPase, TNF-α, and IL-6 levels were measured based on the instructions of their respective commercial kits.

### Assessment of reactive oxygen species

H9c2 cells were plated in 24-well plates. The cell supernatant was discarded after treating the cells with Nar and CoCl_2_ as described previously; each well was then rinsed twice with PBS. Next, using 2,7-dichlorodihydrofluorescein diacetate (DCFH-DA, Cayman Chemical, Michigan, USA), we assessed the intracellular ROS levels. The green fluorescence intensity was proportional to the level of ROS. The wells of each group were added with 500 μl of DCFH staining solution, which was kept at 37°C away from light for 15 min. Afterward, the dye was discarded, and the cells were washed twice with PBS. Finally, fluorescence images were acquired by fluorescence microscopy, and the degree of fluorescence was analyzed by ImageJ software.

### Assessment of mitochondrial membrane potential

As a result of treatment with Nar and CoCl_2_, H9c2 cells were washed twice with PBS and incubated with rhodamine-123 (Rh123, Yuanye Biotechnology, Shanghai, China) at 37°C away from light for 15 min. All experimental steps were completed according to the manufacturer’s requirements. Finally, fluorescence microscopy was used for image acquisition.

### Assessment of apoptosis

Apoptosis levels of H9c2 cells were detected using Hoechst-33258 staining (Solarbio Life Sciences, Beijing, China). Briefly, after drug treatment, cells were incubated with Hoechst-33258 at 37°C for 20 min and followed by two washes with PBS to remove free dye. Afterward, images were collected by using a fluorescence microscope.

### Assessment of Ca^2+^ concentration

Briefly, cells were washed twice with PBS and stained with fluo-3, AM (Life Technologies, Carlsbad, USA) for 15 min at room temperature in the dark environment, followed by two washes with PBS. After that, fluorescence microscopy was used to acquire images.

### Experimental animals

Forty adult male Sprague-Dawley rats weighing 220–250 g, 6–8 eeks, were bought from the Experimental Animal Center of Hebei Medical University (Approval Number: 2103062). Animals are housed under standard laboratory conditions (23 ± 2°C, 50 ± 5% relative humidity, 12 h light/dark cycle). All rats were fed animal maintenance chow (Changsheng biotechnology, Liaoning, China) and provided with purified water available *ad libitum*. After one week of adaptive feeding, one rat per day was used to acutely isolate cardiomyocytes, and the remaining rats were fed normally. All experimental procedures regarding animals were complied with the Guidelines of Animal Experiments from the Ethics Committee of Hebei University of Chinese Medicine.

### Preparation of ventricular cardiomyocytes

Single ventricular myocytes were isolated from 6-8-week-old rats as previously described (Liang et al., 2020). After anticoagulation with intraperitoneal injection of heparin (500 IU/kg), the rats were anesthetized with ethyl carbamate (1 g/kg). The rat heart was then immediately taken out and put in ice-cold Ca^2+^-free Tyrode’s solution containing (in mM): 0.33 NaH_2_PO_4_, 1 MgCl_2_, 5.4 KCl, 10 Glucose, 10 HEPES, and 135 NaCl with a pH of 7.4 adjusted with NaOH. The aorta of the heart is then inserted into the Langendorff device, and the heart was perfused with Ca^2+^-free Tyrode’s solution for 5 min at a 37°C constant temperature device. After that, the heart was perfused with Ca^2+^-free Tyrode’s solution with the addition of type II collagenase (0.6 mg/ml), CaCl_2_ (0.03 mM), Taurine (4.4 mM) and bovine serum albumin (0.5 mg/ml) for 20–25 min. After the heart became soft, the enzymatic fluid in the heart was washed away by Tyrode’s solution without Ca^2+^. The heart was then placed in Krebs buffer (KB) containing (in mM): 25 KH_2_PO_4_, 40 KCl, 80 KOH, 50 Glutamic acid, 10 Glucose, 10 HEPES, 20 Taurine, 1 EGTA, 3 MgSO_4_, and dissected into small pieces. Finally, ventricular myocytes were suspended in KB fluid.

Rats were subcutaneously injected with ISO (85 mg/kg) for two consecutive days to establish a myocardial ischemia model according to previous studies (Allawadhi, Khurana, Sayed, Kumari, & Godugu, 2018; Ghazouani et al., 2019). After that, cells of myocardial ischemia were isolated in the same way as normal rat cardiomyocytes.

### Electrophysiological recording

Isolated cardiomyocytes were investigated under a whole-cell patch clamp to record Ca^2+^ currents. Simply, the pipette puller (Sutter Instruments, Novato, CA, USA) was used to pull a glass pipette with a resistance of 3–5 MΩ filled with (in mM) 20 TEACL, 10 HEPES, 10 EGTA, 5 Mg-ATP, and 120 CsCl with a pH of 7.2 adjusted with CsOH. Myocardial cells were bathed in extracellular solution containing the following compounds (mM): 10 Glucose, 10 HEPES, 1.8 CaCl_2_, 2 MgCl_2_ and 140 TEACL with a pH of 7.4 adjusted with CsOH. This solution allows to reduce the interference from other currents. The electrodes were gently pressed onto the cell surface. The cell was aspirated and formed a high resistance seal at a zero potential difference between the inside and outside of the electrode. Axon patch 200B Amplifier (Axon Instruments, Union City, CA, USA) was filtered at 2 kHz and recorded the Ca^2+^ currents. Analyses of the data were carried out using the pCLAMP 10.7 software (Protocol: duration at a frequency of 10 Hz; the pulse wave form was 10 mV, 1 ms).

### Contractility and Ca^2+^ transient measurement

Cardiomyocyte contractility and Ca^2+^ transients were recorded as described (Guan et al., 2015). Briefly, the cell suspension was gently added dropwise to the perfusion chamber of the inverted microscope. After a few minutes of sedimentation, the cells were perfused with Ca^2+^-free Tyrode’s solution with 1.8 mM CaCl_2_ at a rate of 1 ml/min. The contraction of myocardial cells was measured by the IonOptix Myocam assay system (IonOptix, Milton, MA, USA) and field stimulation was given at 0.5 Hz (duration 2-ms) using a cell stimulator (MyoPacer, IonOptix).

Ca^2+^ transients were detected simultaneously with contraction of the cardiomyocytes. Experimental steps were performed in the dark due to the light sensitivity of the fluorescent Ca^2+^ indicator. Cardiomyocytes were loaded with fluorescent Ca^2+^ indicator fluo2-AM (2 mM) for 10 min. Cells were stimulated with a field intensity of 0.5 Hz and passed alternately through 340 and 380 nm filters (bandwidth of ±15 nm). Finally, the emitted fluorescence was observed at 510 nm.

### Data analysis and statistics

Data were processed and analyzed using Origin 7.5 software (OriginLab Corp.). Statistical analysis of the data was conducted using one-way analysis of variance (ANOVA) followed by Bonferroni correction for multiple comparisons. Data were expressed as the means ± SEM. *p* < 0.05 was considered statistically significant. Clampfit 10.7 (Molecular Devices) was used to analyze the I_Ca-L_ data, and Boltzmann functions were used to fit the steady-state activation and inactivation of I_Ca-L_.

## Results

### Effects of nar on MI were explored based on network pharmacology

ADME-related information of Nar was searched by TCMSP. OB is the oral bioavailability of the drug and DL is the similarity of the drug, which are important for the screening and evaluation of the drug. The OB and DL of Nar were 59.29% and 0.21, respectively, which met the lower limit.

**FIGURE 2:**
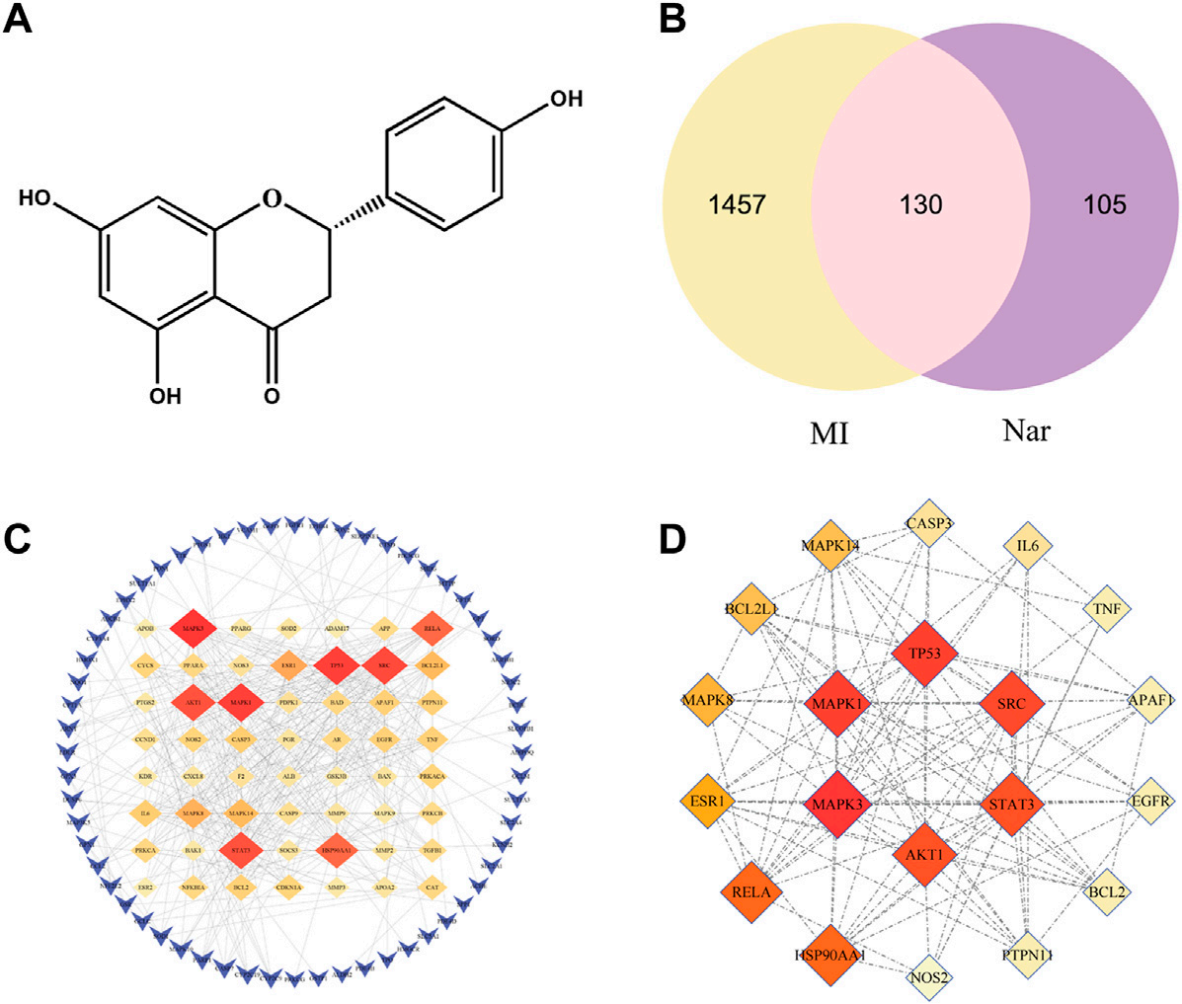
Venn diagram and PPI network. **(A)** Chemical structure of naringenin (Nar). **(B)** Venn diagram of the intersection target between MI and Nar. **(C)** PPI network with 130 intersecting targets. **(D)** Top 20 targets of PPI network.

The molecular structure formula of Nar is presented in Figure 2A (C_15_H_12_O_5_, MW:272.27). A total of 235 targets of Nar were obtained from TCMSP, CTD, PharmMapper and Swiss TargetPrediction databases. Meanwhile, a total of 1,587 targets of MI were obtained from GeneCards, DisGeNET and OMIM databases. The Venn diagram showed that 130 potential targets had relationships with Nar and MI (Figure 2B). To obtain the naringenin target network, the intersection targets were imported into the string database, and the interaction score was set to greater than 0.9. The graphs were generated using Cytoscape software (Figure 2C). A darker color and a larger diameter of the nodes in the graph represent a larger degree value. The top 20 core target proteins were presented in Figure 2D according to the degree value in the PPI network.

**FIGURE 3:**
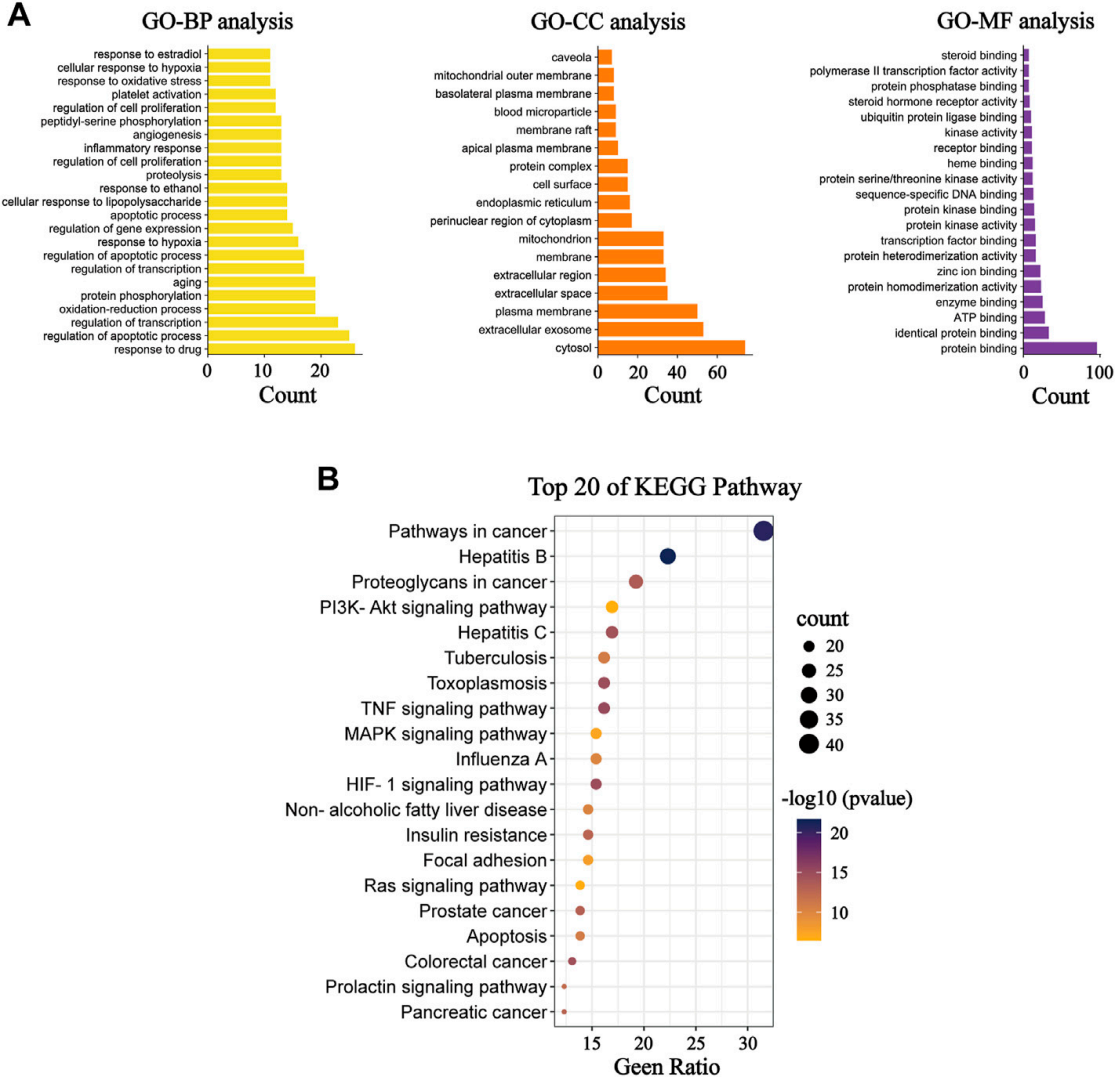
GO and KEGG functional analysis. **(A)** Diagram of GO BP analysis; diagram of GO CC analysis; and diagram of GO MF analysis. **(B)** KEGG pathway enrichment analysis.

Functional bioinformatics analysis was further performed to better elucidate the mechanism of action of Nar in the treatment of MI. Specifically, we found that the biological processes (BP), cellular component (CC), and molecular function (MF) consisted of 241, 17, and 31 items, respectively. BP analysis showed that most of these targets are closely related to the regulation of apoptotic processes, redox, response to hypoxia, inflammatory response, regulation of cell proliferation, and oxidative stress response. CC analysis indicated that these targets were distributed in the cytosol, mitochondria, and endoplasmic reticulum. MF analysis revealed that these targets were associated with protein binding, ATP binding and enzyme binding. KEGG enrichment analysis suggests that the majority of pathways mentioned here are closely related to apoptosis and inflammation (Figure 3). The results show that apoptosis, mitochondrial energy metabolism, inflammation, and oxidative stress are all involved in Nar preventing MI.

### Effects of nar on CoCl_2_-induced damage in H9c2 cells

**FIGURE 4:**
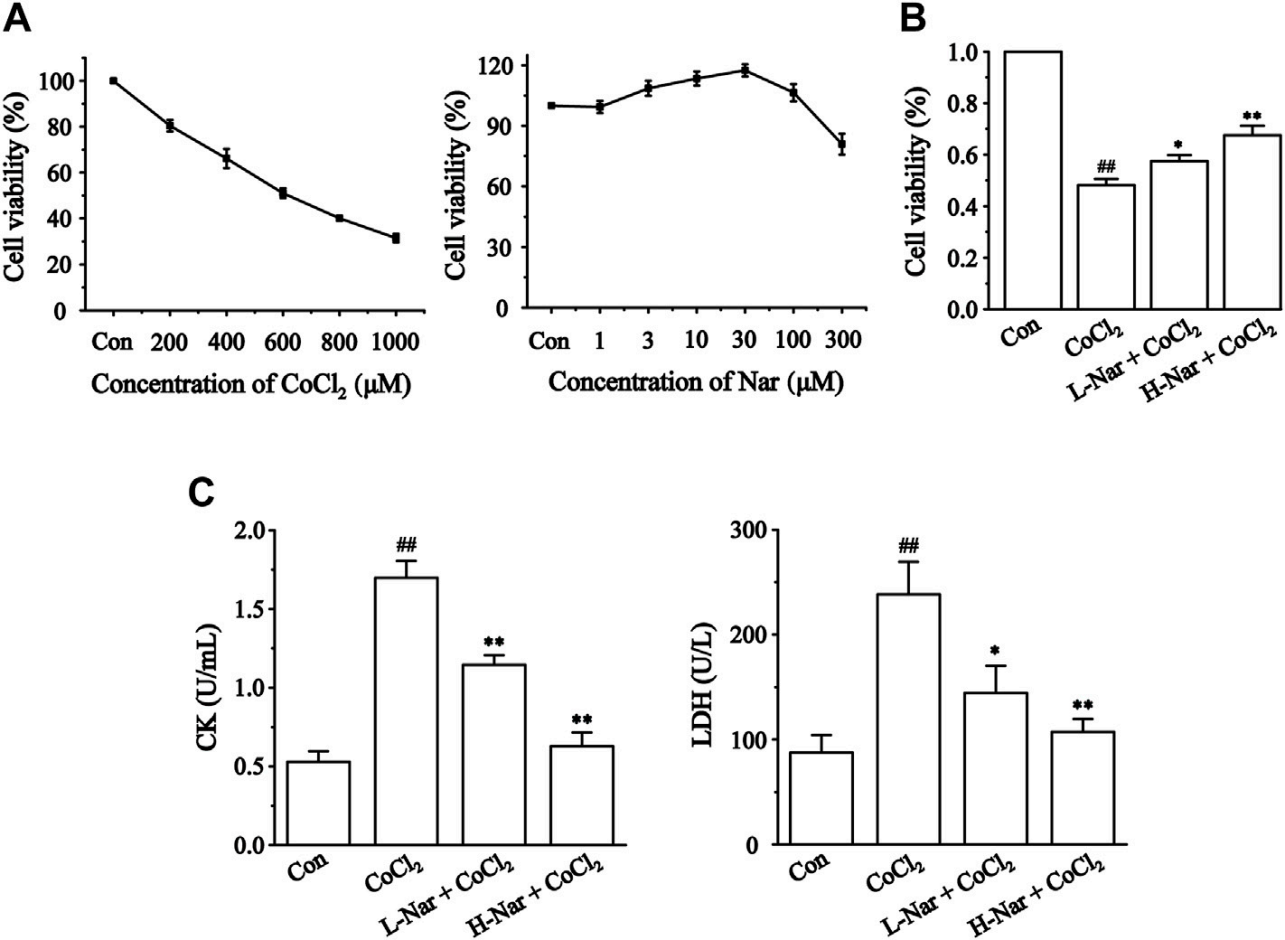
Effects of CoCl_2_ and Nar on H9c2 cells viability, and the effects of Nar on CoCl_2_-induced cardiomyocyte injury. Viability was measured by CCK-8. **(A)** H9c2 cells were incubated with CoCl_2_ (200–1,000 μM) alone and Nar (1–300 μM) alone for 24 h. **(B)** Cells were pretreated with Nar (10 and 30 μM) for 4 h and then exposed with CoCl_2_ (600 μM) for 24 h. **(C)** The effects of Nar on the release amount of CK and LDH in H9c2 cells. Data were expressed as the mean ± SEM of each group. ^##^ *p* < 0.01 vs. Con group; ^*^ *p* < 0.05, ^**^ *p* < 0.01 vs. CoCl_2_ group, n = 6 each group. Note: L-Nar: 10 μM and H-Nar: 30 μM.

The protective effect of Nar was initially assessed based on the survival of H9c2 cells induced by CoCl_2_. Nar itself had almost no cytotoxic effect on H9c2 cells in the concentration range of 1–100 μM; the strongest protective effect on cells was at 30 μM of Nar (Figure 4A). Therefore, 10 or 30 μM doses of Nar were selected for further experiments. Figure 4B displays that the survival rate of H9c2 cells was dramatically decreased in the CoCl_2_ group versus the Con group (*p* < 0.01). Treatment of H9c2 cells with 10 or 30 μM Nar attenuated 600 μM CoCl_2_-induced cell damage (*p* < 0.05 or 0.01).

We assayed the release of CK and LDH in cells (Figure 4C) to further verify the protective effect of Nar. The levels of CK and LDH were markedly increased versus the Con group after CoCl_2_ treatment (*p* < 0.01), whereas Nar treatment reduced the release of CK and LDH versus the CoCl_2_ group (*p* < 0.01 or 0.05).

### Effects of nar on oxidative stress levels

**FIGURE 5:**
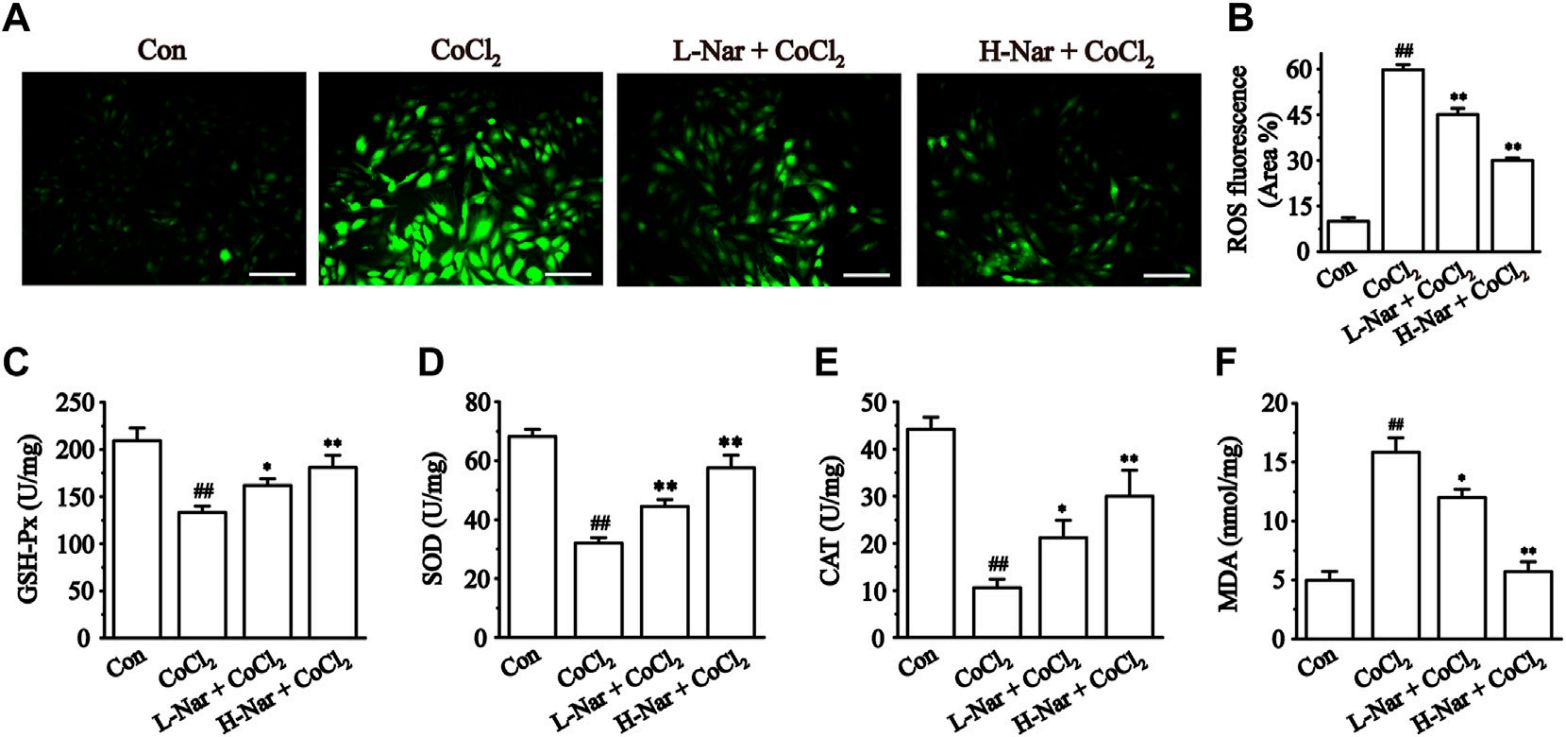
Effects of Nar on CoCl_2_-induced the levels of ROS, and the effects of Nar on the levels of oxidative stress in H9c2 cells. **(A)** Morphological changes of intracellular ROS (scale bar: 100 μm; magnification: ×200). **(B)** Statistical analysis of ROS fluorescence. **(C)** GSH-Px activity. **(D)** SOD activity. **(E)** CAT level and **(F)** MDA level. Data were expressed as the mean ± SEM of each group. ^##^ *p* < 0.01 vs. Con group; ^*^ *p* < 0.05, ^**^ *p* < 0.01 vs. CoCl_2_ group, n = 6 each group. Note: L-Nar: 10 μM and H-Nar: 30 μM.

Myocardial ischemia leads to the production of large amounts of ROS. It was found that the fluorescence intensity of ROS was obviously enhanced after CoCl_2_ treatment (*p* < 0.01), but the fluorescence level was significantly reduced after pretreatment with different doses of Nar (*p* < 0.01) (Figures 5A,B).

We next measured the intracellular levels of GSH-P_X_, SOD, CAT, and MDA. The concentrations of GSH-P_X_, SOD, and CAT were markedly reduced after exposure to CoCl_2_ (*p* < 0.01), but these parameters were reversed after pretreatment with Nar (*p* < 0.01 or 0.05). Meanwhile, the content of MDA was increased after exposure with CoCl_2_ (*p* < 0.01). The MDA level in H9c2 was reduced after pretreatment with Nar (*p* < 0.01 or 0.05) (Figures 5C–F). These results demonstrate that Nar can remarkably reduce the level of oxidative stress in cells.

### Effects of nar on mitochondrial function

**FIGURE 6:**
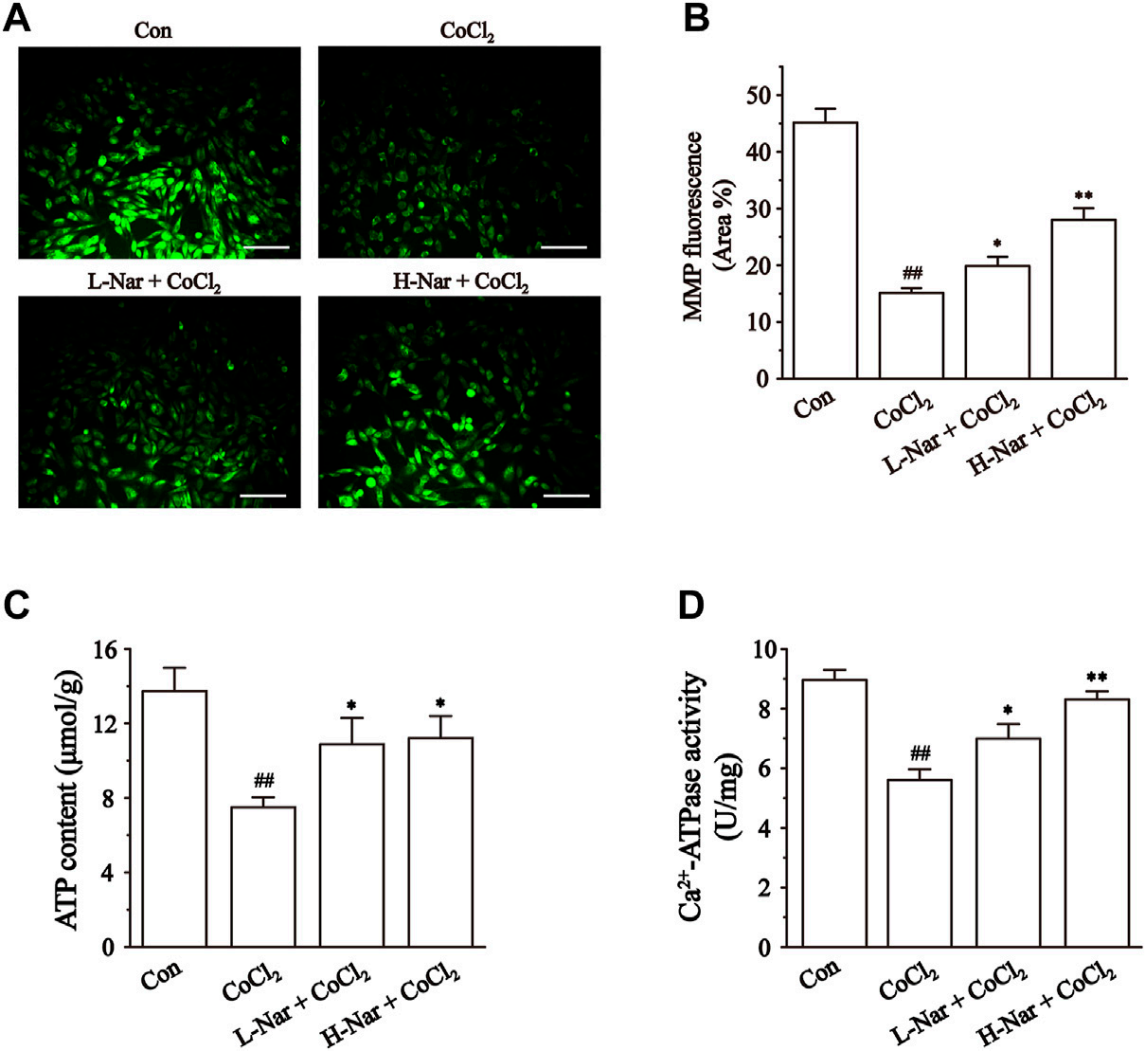
Effects of Nar on CoCl_2_-induced the mitochondrial damage in H9c2. **(A)** Morphological changes of mitochondria were observed by rhodamine 123 (scale bar: 100 μm; magnification: ×200). **(B)** Statistical analysis of mitochondrial fluorescence was presented. **(C)** ATP content, and **(D)** Ca^2+^-ATPase activity were detected. Data were expressed as the mean ± SEM of each group. ^##^ *p* < 0.01 vs. Con group; ^*^ *p* < 0.05, ^**^ *p* < 0.01 vs. CoCl_2_ group, n = 6 each group. Note: L-Nar: 10 μM and H-Nar: 30 μM.

Mitochondrial dysfunction is closely associated with myocardial ischemia (Messadi et al., 2014). We evaluated the state of mitochondria by detecting the uptake of the fluorescent dye Rh123 by mitochondria (Figures 6A,B). After CoCl_2_ treatment, lower levels of green fluorescence were observed in H9c2 cells. (*p* < 0.01), however, the fluorescence intensity was significantly enhanced after pretreatment with Nar. The results indicated that Nar can maintain the integrity of mitochondria.

Impaired mitochondrial function leads to reduced ATP production (Lopaschuk et al., 2010). Ca^2+^-ATPase activity is an indirect reflection of the change in ATP content (Lu et al., 2020). After pretreatment with Nar, the level of ATP and the activity of Ca^2+^-ATPase were increased to different extents in H9c2 cells (*p* < 0.1 or 0.5). However, the levels of ATP and Ca^2+^-ATP in the cells were significantly decreased after CoCl_2_ treatment (*p* < 0.1) (Figures 6C,D).

### Effects of nar on inflammation

**FIGURE 7:**
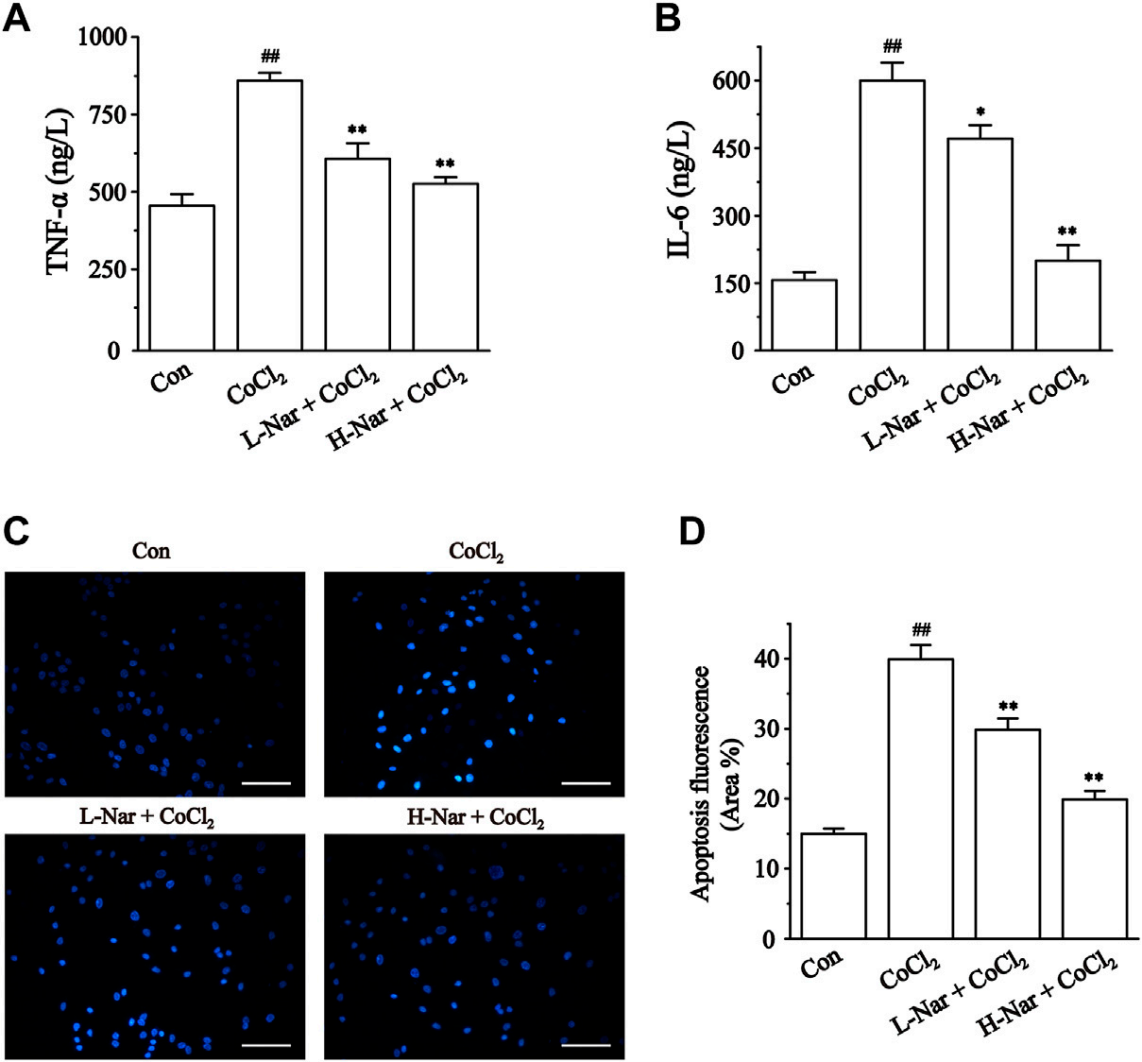
Effects of Nar on CoCl_2_-induced inflammation and apoptosis in H9c2. **(A)** TNF-α and **(B)** IL-6 were detected by ELISA. **(C)** Cardiomyocyte apoptosis was assessed by Hoechst 33258 staining (scale bar: 100 μm; magnification: ×200). **(D)** Statistical analysis of apoptosis fluorescence was presented. Data were expressed as the mean ± SEM of each group. ^##^ *p* < 0.01 vs. Con group; ^*^ *p* < 0.05, ^**^ *p* < 0.01 vs. CoCl_2_ group, n = 6 each group. Note: L-Nar: 10 μM and H-Nar: 30 μM.

To identity the anti-inflammatory effects of Nar in H9c2, we next evaluated the levels of IL-6 and TNF-α (Figures 7A,B). IL-6 and TNF-α levels were significantly increased in CoCl_2_-treated H9c2 cells (*p* < 0.01). However, IL-6 and TNF-α levels were significantly decreased after pretreatment with Nar (*p* < 0.01 or 0.05).

### Effects of nar on apoptosis

Ischemia and hypoxia eventually cause apoptosis in cardiac myocytes. These results indicated that the blue fluorescence level of H9c2 cells was enhanced after treatment with CoCl_2_ (*p* < 0.01). But the fluorescence intensity was notably diminished after pretreatment with Nar (*p* < 0.01 or 0.05) (Figures 7C,D).

### Effects of nar on intracellular Ca^2+^ concentrations

**FIGURE 8:**
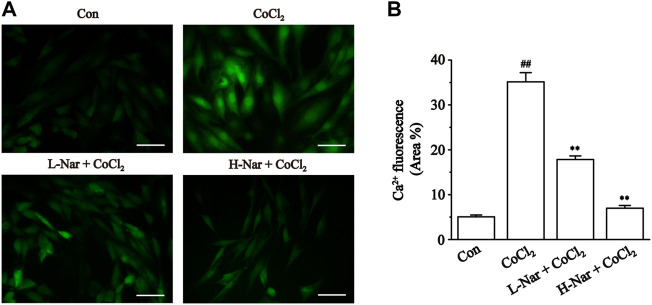
Effects of Nar on CoCl_2_-induced the levels of Ca^2+^ concentration in H9c2. **(A)** Morphological changes of intracellular Ca^2+^ as observed by Fluo-3 staining (scale bar: 50 μm; magnification: ×400). **(B)** Statistical analysis of ROS fluorescence was presented. Data were expressed as the mean ± SEM of each group. ^##^ *p* < 0.01 vs. Con group; ^*^ *p* < 0.05, ^**^ *p* < 0.01 vs. CoCl_2_ group, n = 6 each group. Note: L-Nar: 10 μM and H-Nar: 30 μM.

We confirmed that Nar reduces the increase of Ca^2+^ concentration in H9c2 cells. The fluorescence intensity of Ca^2+^ was significantly enhanced after CoCl_2_ treatment (*p* < 0.01), however, the levels of fluorescence were markedly reduced after pretreatment with different doses of Nar (*p* < 0.01) (Figure 8). It was shown that Nar can reduce intracellular Ca^2+^ concentrations.

### Verification of I_Ca-L_ in isolated cardiomyocytes

**FIGURE 9:**
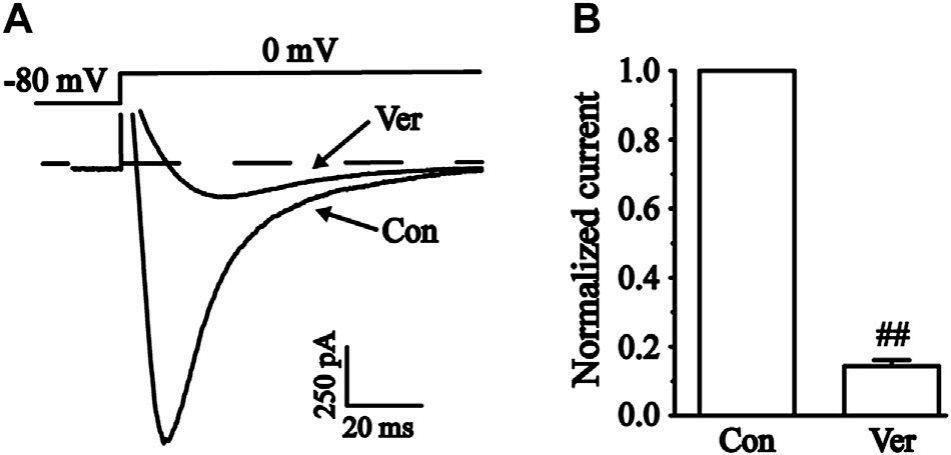
Verification of L-type calcium current (I_Ca-L_) in isolated cardiomyocytes. **(A)** Representative traces of I_Ca-L_ with a steady-state activation before and after using 10 μM Ver. **(B)** Pooled data were expressed as the mean ± SEM. Each group has 10 cells from five to six rat hearts. ^##^ *p* < 0.01 vs. Con group.

Two types of voltage-dependent calcium channels (L-type and T-type) are present in cardiac myocytes. T-type calcium currents are negligible in most ventricular myocytes, so calcium currents are usually referred to as L-type here (Bers, 2002). The L-type calcium channel blocker verapamil (Ver, 10 μM) is almost completely effective at blocking the currents (Figure 9). These results indicated that the current recorded in ventricular myocytes was the I_Ca-L_.

### Effects of nar on I_Ca-L_ in normal and ischemic ventricular myocytes

**FIGURE 10:**
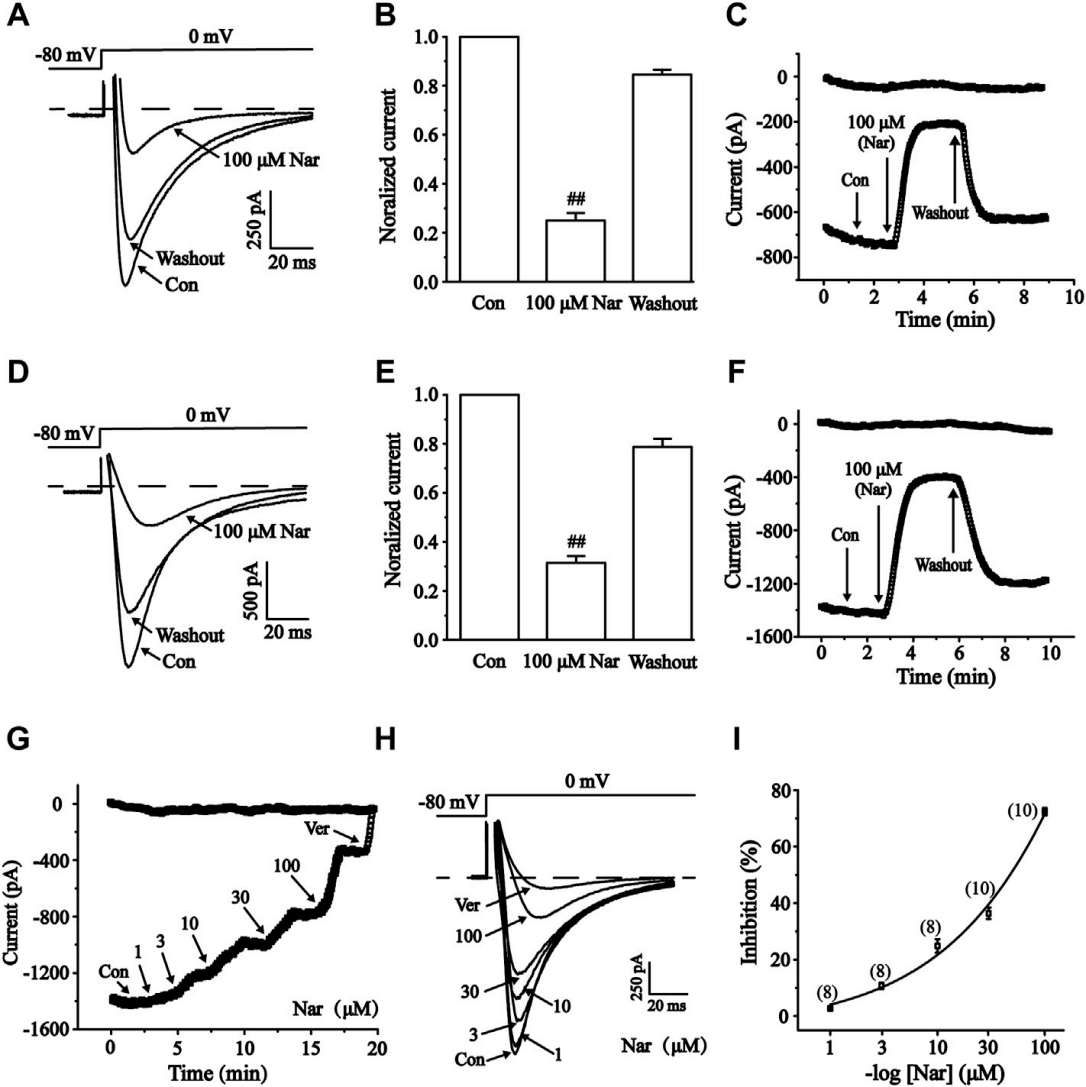
Reversible effects of Nar on I_Ca-L_ in normal and ischemic ventricular myocytes. The corresponding letters and numbers are recorded on the main graphs to indicate the time. **(A–C)** Effects of Nar on I_Ca-L_ in normal cardiomyocytes. **(D–F)** Effects of Nar on I_Ca-L_ in ischemic cardiomyocytes. **(A,D)** Representative examples of I_Ca-L_, **(B,E)** summarized data and **(C,F)** time constant of I_Ca-L_ were documented under Con, Nar (100 μM) and washout conditions. Values were expressed as the mean ± SEM. Each group has 10 cells from five to six rat hearts. ^##^ *p* < 0.01 vs. Con group. During the periods indicated by horizontal lines, cells were continuously perfused with 1, 3, 10, 30 and 100 μM Nar and 10 μM Ver. **(G)** Time constants and **(H)** typical traces were recorded. **(I)** The percentage inhibition of Nar was expressed as concentration-response curve, each group has eight to 10 cells from seven rat hearts.

I_Ca-L_ was measured under control conditions, followed by exposure to 100 μM Nar and washout (Figures 10A–F). Nar (100 μM) greatly inhibited I_Ca-L_ in normal and ischemic cells with inhibition rates of 75.0 ± 3.10% and 68.6 ± 2.70%, respectively (*p* < 0.01). I_Ca-L_ was restored after washing off Nar with external solutions, thus suggesting that the effect of Nar on I_Ca-L_ was not caused by running down. The present results indicate that the effect of Nar on Ca^2+^ currents is largely reversible.

It was been found that Nar inhibited I_Ca-L_ in a concentration-dependent manner (Figures 10G–I). Logistic equation was used to fit the concentration-response curve: *y* = *A*_*2*_ + (*A*_*1*_—*A*_*2*_)/[1 + (*x*/*x*_*0*_)^*p*^]. Here, *y* is the response, *A*_*1*_ and *A*_*2*_ are the maximum and minimum response, respectively, *x* is the drug concentration, and *p* is the Hill coefficient. The inhibition of I_Ca-L_ in myocardial cells (n = 8-10) treated with 1, 3, 10, 30, and 100 μM Nar were 2.50 ± 0.31%, 13.14 ± 2.18%, 23.22 ± 2.67%, 34.55 ± 2.59%, and 75.10 ± 3.07%, respectively. The half-maximal inhibition (IC_50_) was 41.493 μM.

### Effects of nar on current-voltage (I-V) relationship of I_Ca- L_

**FIGURE 11:**
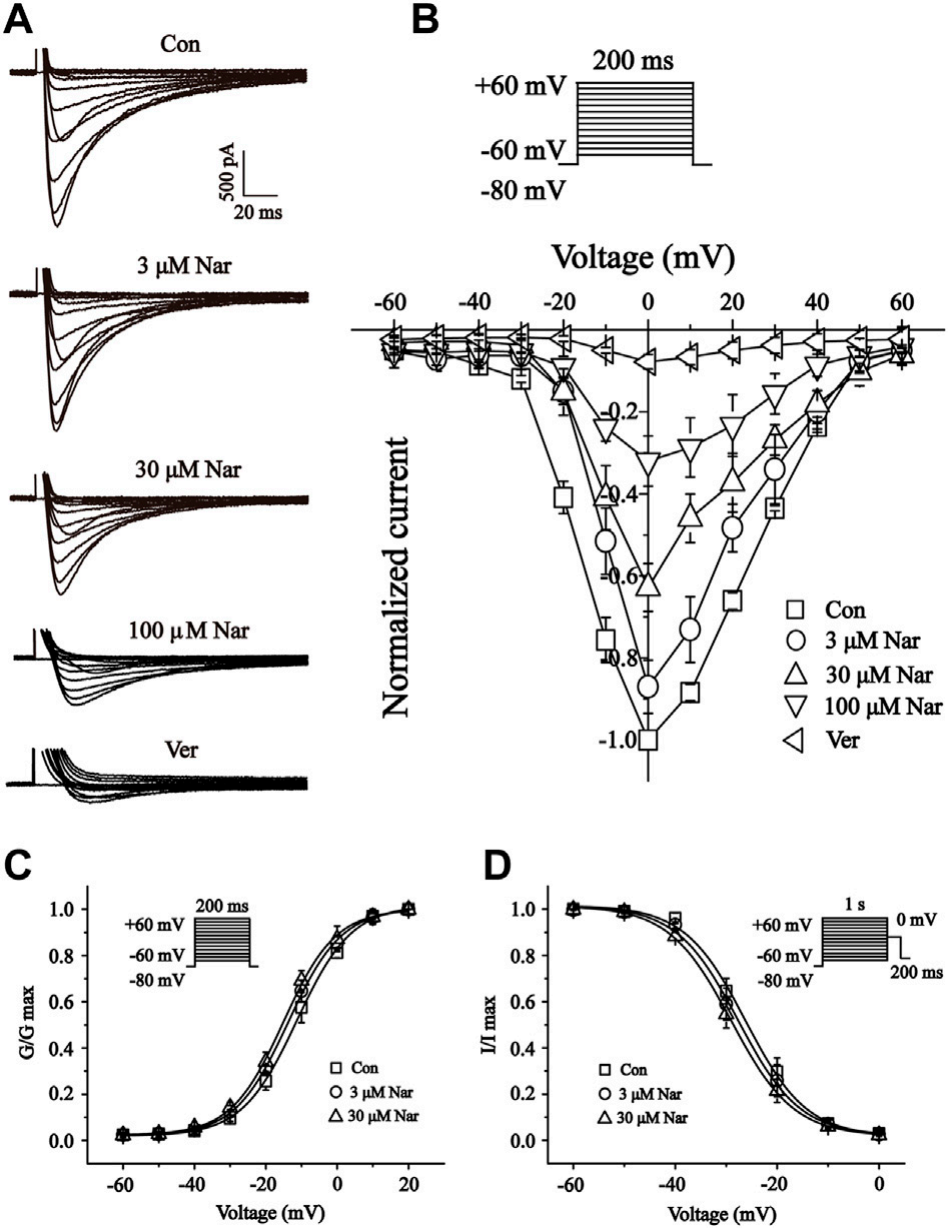
Effects of Nar on the current-voltage relationship (I-V curve) of I_Ca-L_, and the effects of Nar on activation and inactivation properties of I_Ca-L_. **(A)** Representative recordings of I_Ca-L_ from isolated cardiomyocytes treated with Con, Nar (3, 30 and 100 μM) and Ver (10 μM) conditions. **(B)** I-V curve of I_Ca-L_ with Con, Nar (3, 30 and 100 μM) and Ver (10 μM) conditions. Voltage dependence of **(C)** ICa-L activation and **(D)** steady-state inactivation in the Con and Nar (3 and 30 μM) conditions. Values were expressed as the mean ± SEM, each group has eight to 10 cells from seven rat hearts.

In single myocardial cells, the I_Ca-L_ was recorded with a steady-state activation protocol along with control, Nar (3, 30 and 100 μM) and Ver conditions (Figure 11A). Current-voltage (I-V) curves of I_Ca-L_ under the control condition, the presence of different concentrations of Nar and Ver condition was shown in Figure 11B. In the I-V relationship, the amplitude of the current is significantly enhanced in the voltage range between -20 and +20 mV. However, the reverse potential and current-voltage relationship of I_Ca-L_ had no significant variation.

### Effects of nar on steady-state activation and inactivation of I_Ca- L_

We further documented the activation and inactivation gating properties of Ca^2+^ channels in the absence and presence of Nar (3 and 30 μM) (Figures 11C,D). Values at V_1/2_ for the normalized activation curves were -11.30 ± 0.40 mV with a slope factor (k) of 7.69 ± 0.37 mV for control, -13.72 ± 0.34 mV with a k value of 7.49 ± 0.32 mV for Nar of 3 μM, and -15.11 ± 0.41 mV with a k value of 7.34 ± 0.37 mV for Nar of 30 µM. Values of V_1/2_ for the steady-state inactivation were -26.17 ± 0.79 mV with a k value of 5.99 ± 0.72 mV for control, -27.64 ± 0.73 mV with a k value of 6.03 ± 0.68 mV for Nar of 3 μM, and -29.00 ± 0.34 mV with a k value of 6.11 ± 0.32 mV for Nar of 30 µM. The results showed that Nar had no significant effect on steady-state activation and inactivation of I_Ca-L_.

### Effects of nar on myocyte contractility

**FIGURE 12:**
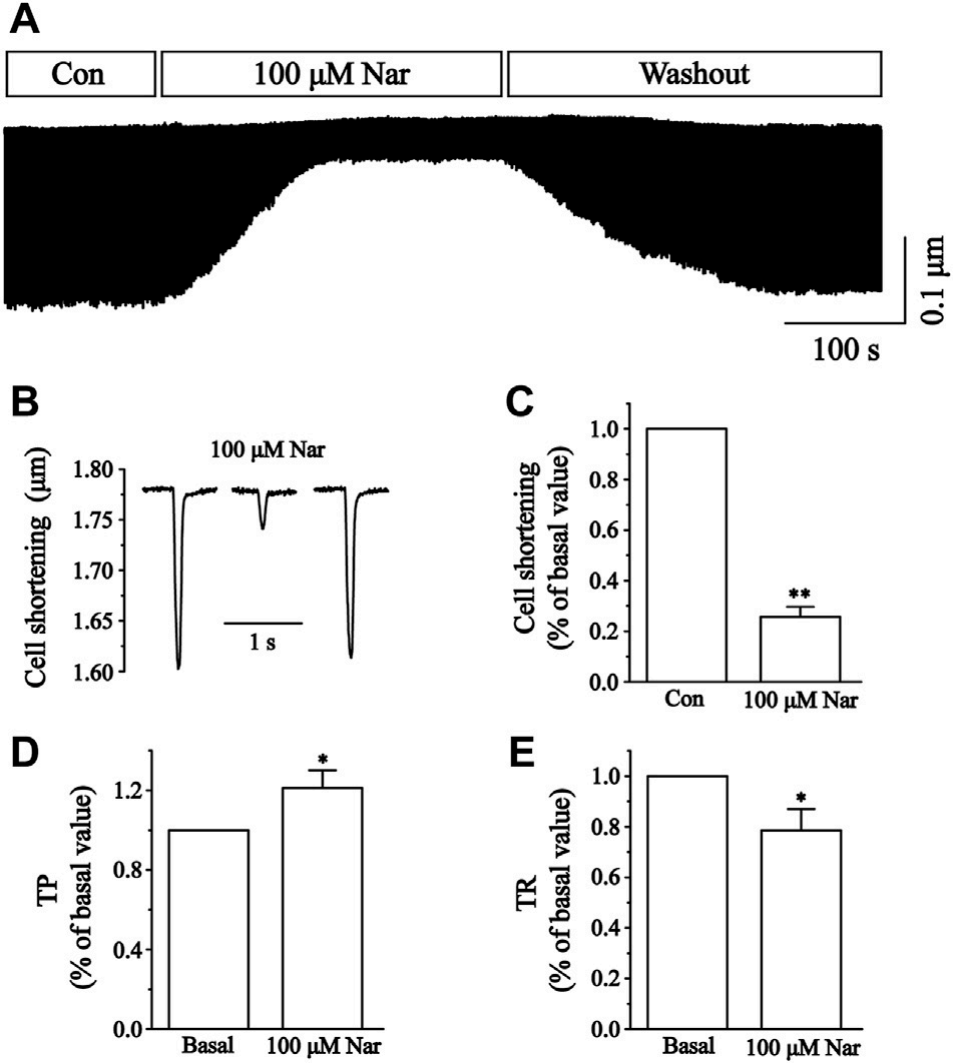
Effects of Nar on Ca^2+^ contraction in isolated cardiomyocytes and time parameters of cell shortening. **(A)** Recordings of cardiomyocyte contraction on time course under the Con, Nar (100 μM) and washout conditions. **(B)** Single typical recordings of cell shortening at Con, Nar (100 μM) and washout. **(C)** Summarized data at Con and Nar (100 μM). Values were expressed as the mean ± SEM. Each group has 9 cells from five rat hearts. ^##^ *p* < 0.01 vs. Con group; ^**^ *p* < 0.01 vs. Con group. **(D,E)** Summarized data of TP and TR at Con and Nar (100 μM). Values were expressed as the mean ± SEM (n = 6 cells). Each group has 6 cells from three rat hearts. ^#^ *p* < 0.05 vs. Basal; ^*^ *p* < 0.05 vs. Basal.

We next determined the effects of 100 μM Nar on the contractility of cardiomyocytes. The representative track of cell shortening before and after administration of Nar (100 µM) was presented in Figure 12A. The inhibition rate of Nar was 74.22 ± 3.09% for 100 μM (*p* < 0.01) (Figure 12C). The time to 50% of the peak (TP) represents the rate of myocyte shortening or Ca^2+^ elevation, and the time to 50% of the baseline (TR) represents cellular relaxation or Ca^2+^ reuptake. Compared to the Basal, 100 µM of Nar enhanced the TP by 21.64 ± 8.83%, and decreased the TR by 21.43 ± 8.43% (*p* < 0.05) (Figures 12D,E).

### Effects of nar on cell Ca^2+^ transients

**FIGURE 13:**
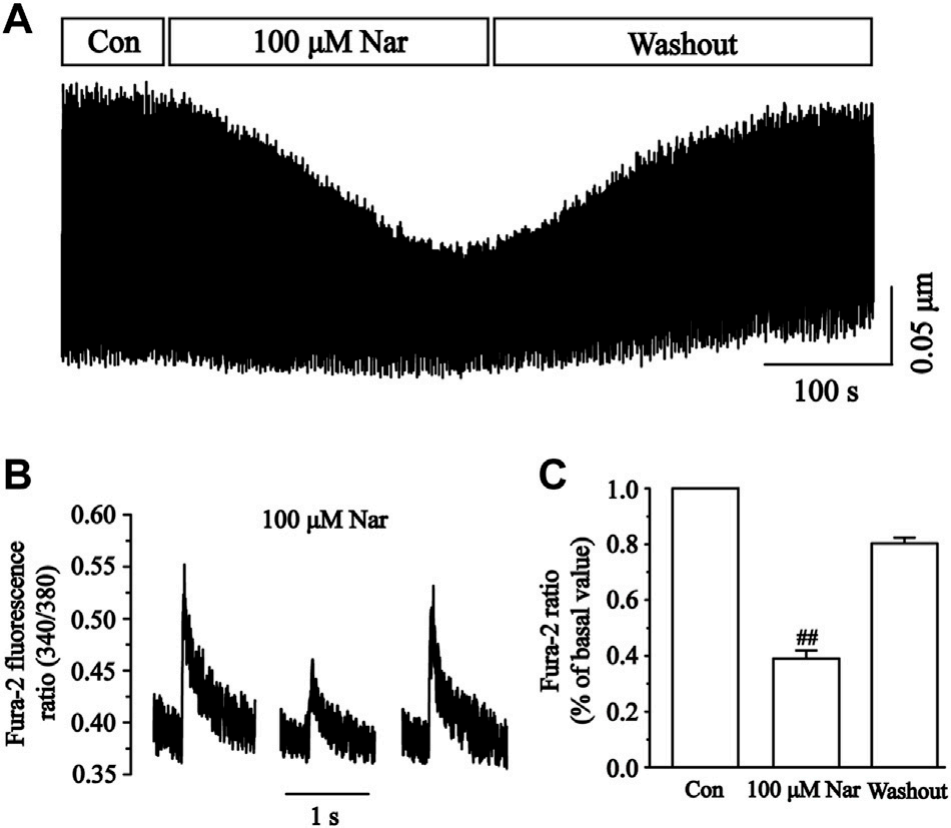
Effects of Nar on Ca^2+^ transients in isolated cardiomyocytes. **(A)** Recordings of Ca^2+^ transients on time course under the Con, Nar (100 μM) and washout conditions. **(B)** Single typical recordings of Ca^2+^ transients at Con, Nar (100 μM) and washout. **(C)** Summarized data at Con, Nar (100 μM) and washout. Values were expressed as the mean ± SEM, each group has 9 cells from five rat hearts.

Changes in cellular Ca^2+^ transients before and after the administration of Nar are presented in Figure 13. The inhibition of Ca^2+^ transients by Nar of 100 μM was 61.00 ± 2.91% (*p* < 0.01). The results showed that Nar partially and reversibly reduced the transient of Ca^2+^ currents in cardiomyocytes.

## Discussion

Currently, ischemic heart disease has become a huge threat to human health and the global economy (H. Yu, Lu, Zhu, & Wang, 2017). Naringenin is a naturally occurring major flavonoid, abundant in the peel of citrus fruits that has various biological properties such as cardioprotective effects (Testai et al., 2017). Here, the mechanism of action of Nar against MI was systematically demonstrated through a combination of network pharmacological analysis and experimental validation.

Myocardial ischemia is a very complex pathophysiological process that involves multiple biological mechanisms. A total of 235 potential targets for Nar and 1,587 targets related to MI were obtained through online public databases. 130 intersection targets were obtained *via* the Venn analysis tool to perform enrichment analysis. The top 10 core targets for Nar treatment of MI are MAPK3, TP53, SRC, AKT1, STAT3, HSP90AA1, RELA, ESR1and MAPK8 (degree >17). Of these, HSP90AA1 can regulate mitochondrial function to protect the heart (Budas, Churchill, Disatnik, Sun, & Mochly-Rosen, 2010). ESR1 is well known to protect against myocardial ischemia/reperfusion injury by inhibiting MPTP openings (Kabir et al., 2015). Meanwhile, MAPK and TP53 are highly expressed in the Ca^2+^ signaling pathway enriched by KEGG. Biological enrichment analysis indicates that Nar improves apoptosis, mitochondrial energy metabolism, inflammation and oxidative stress. Mitochondrial dysfunction activates the Ca^2+^ transport system and Ca^2+^ overload drives the opening of MPTP, thus leading to cellular inflammation and apoptosis (B. Zhou & Tian, 2018). Therefore, calcium signals play an essential role in the mechanism of naringenin against myocardial ischemia.

Based on the results of network pharmacology enrichment analysis, this study verified the effects of Nar on apoptosis, impaired mitochondrial energy metabolism, inflammation and oxidative stress damage using H9c2 cardiomyocytes. The H9c2 cell line is derived from the rat embryonic heart, which has similar electrophysiological and biochemical characteristics of cardiomyocytes (Fang, Luo, & Luo, 2018). Ischemia directly causes hypoxic damage to cardiomyocytes, resulting in apoptosis, structural changes and functional decline, which are closely associated with a variety of diseases (Munoz-Sanchez & Chanez-Cardenas, 2019; Veldhuizen et al., 2022). CoCl_2_ is a common chemical drug used to induce hypoxic injury because of its ease of use and stable physicochemical properties (Munoz-Sanchez & Chanez-Cardenas, 2019). Co^2+^ in CoCl_2_ can replace iron ions in the oxygen sensor (porphyrin ring of hemoglobin) preventing hemoglobin from binding to oxygen, thus simulating a hypoxic environment and producing a series of reactions similar to those under hypoxic conditions. Compared to low oxygen-induced hypoxia and the use of other hypoxia mimics, CoCl_2_ can strongly stabilize hypoxia-inducible factor (HIF)-1α and HIF-2α under normoxic conditions, which can last for several hours (Munoz-Sanchez & Chanez-Cardenas, 2019). Therefore, using the CoCl_2_-induced hypoxia model under normoxic conditions provides us with more time to analyze the sample.

CoCl_2_-induced H9c2 cell injuries are a reliable method to simulate hypoxic damage to cardiomyocytes *in vitro* (Cheng et al., 2017). The present study showed that Nar reduced H9c2 cell injury as manifested by a reduction in the release of CK and LDH (Figure 4). Myocardial hypoxia-induced mitochondrial damage generates large amounts of reactive oxygen species and superoxide, leading to adverse stimuli such as oxidative stress and Ca^2+^ overload, and further causing apoptosis and necrosis. Ischemia-induced ETC alterations are the main cause of the overproduction of ROS during reperfusion (Hausenloy & Yellon, 2013; Bugger & Pfeil, 2020). A large amount of ROS and Ca^2+^ overload trigger the loss of mitochondrial membrane potential, leading to the release of mitochondrial proteins and the inability to produce ATP (Bugger & Pfeil, 2020). These proteins, including cytochrome C, apoptosis-inducing factor, and others, can promote apoptosis in cardiomyocytes (Di Lisa, Carpi, Giorgio, & Bernardi, 2011). Damage to mitochondrial DNA and/or overproduction of ROS has been shown to induce inflammation (Oka et al., 2012). It was found that Nar decreased the content of ROS and lipid peroxide MDA and enhanced the activity of antioxidant enzymes in CoCl_2_-induced H9c2 cells (Figure 5). Meanwhile, Nar reversed the significant decrease in MMP levels as well as ATP content and Ca^2+^-ATPase activity (Figure 6). Nar also significantly reduced the levels of inflammatory factors (such as TNF-α and IL-6) and apoptosis. (Figure 7). The present studies are consistent with those revealed by GO and KEGG enrichment analysis.

The ubiquitous second messenger Ca^2+^ is essential in the electrical activity of the heart and regulates a variety of physiological activities such as pacing, excitation-contraction coupling, and neurotransmitter release (Hofmann, Flockerzi, Kahl, & Wegener, 2014). Here, studies by us found that Nar significantly inhibited Ca^2+^ concentration in H9c2 cells (Figure 8). Currently, it is often said that the Ca^2+^ current in cardiac myocytes mainly refers to the I_Ca-L_ generated by the opening of L-type Ca^2+^ channels (Bers, 2002). Ca^2+^ overload in pathological conditions is strongly associated with arrhythmias, cardiac hypertrophy and heart failure (Markandeya et al., 2015; Santulli, Xie, Reiken, & Marks, 2015; Zhang et al., 2021). Therefore, intracellular Ca^2+^ homeostasis is important for the maintenance of normal heart physiological function (Barry & Bridge, 1993). The current study showed that Nar inhibited L-type Ca^2+^ currents in a concentration-dependent manner, and this effect is partially reversible without affecting the steady-state activation and inactivation of I_Ca-L_ (Figure 10, Figures 11C,D). The activation curve reflects the speed and ease of Ca^2+^ channel opening, and the inactivation curve reflects the process of channel closure. The current results show that Nar has no effect on channel dynamics. However, whether channel proteins affect the amplitude of Ca^2+^ currents will be investigated in future studies. Also, Nar has not been shown to affect the I-V relationship or the reversal potential of I_Ca-L_ (Figures 11A,B). The results suggest that Ca^2+^ currents inhibited by Nar we present in ventricular myocytes are due to the influence of Ca^2+^ channels.

Calcium-regulated excitation-contraction coupling controls the contractile function of cardiac myocytes. Cardiomyocyte contractile excitability causes the opening of voltage-gated L-type calcium channels (LTCCs), which allows Ca^2+^ to enter the cytoplasm and bind to receptors on RyR2, thus triggering the release of large amounts of Ca^2+^ from the SR (Gorski, Ceholski, & Hajjar, 2015; Lahiri, Aguilar-Sanchez, & Wehrens, 2021). We found that contractility and Ca^2+^ transients in cardiomyocytes are significantly diminished in the presence of Nar (Figures 12A–C, Figure 13). However, it was realized that Nar has more significant effects on cell contraction than Ca^2+^ transients. One possible reason for this phenomenon is that cell contraction is a complex process related not only to the concentration of intracellular Ca^2+^, but also to the proteins involved in contraction or regulation (e.g., actin, myosin and troponin) (Adamcova et al., 2006). Future work will investigate more detailed factors underlying the effects of Nar on myocardial contractility. TP and TR are representative parameters describing the rate of myocardial contraction and relaxation. It was found that TP was elevated while TR was reduced in the presence of 100 µM Nar (Figures 12D,E). The current results suggest that Nar reduces the rate of contraction and thus indirectly inhibits Ca^2+^ concentration, as evidenced by a transient decrease in Ca^2+^.

Calcium overload is thought to be a predisposing cause of myocardial ischemia (Cao et al., 2006). Myocardial contractility and contraction speed are among the important factors affecting myocardial oxygen consumption. Decreased myocardial oxygen consumption following decreased contractility is one of the important mechanisms in the treatment of myocardial ischemia (Crystal, Silver, & Salem, 2013). Here, Nar significantly inhibited L-type Ca^2+^ currents, cell contractility, and Ca^2+^ transients. These inhibitory effects are essential mechanisms for Nar protection against myocardial ischemic damage.

The current study was devoted to exploring the protective effects and mechanisms of Nar against MI, mainly focusing on calcium channels. However, it cannot be excluded that Nar affects other ion channels in the long term. Previous studies have shown that Nar activates mitochondrial potassium channels in dermal fibroblasts and blocks HERG potassium channels in *Xenopus oocytes* (Scholz et al., 2005; Kampa et al., 2019). In addition, it was reported that Nar inhibited NaV1.8 voltage-gated sodium channels in rat dorsal root ganglion neurons (Y. Zhou et al., 2019). Therefore, further studies are needed in the future to explore the effects of Nar on other ion channels.

## Conclusion

This work is to systematically explore the potential targets of Nar for the treatment of MI using network pharmacology. It was demonstrated that Nar attenuated MI-induced oxidative stress, mitochondrial dysfunction, inflammation and apoptosis. Most importantly, the current study indicated that Nar exhibited cardioprotective properties by inhibiting LTCCs thereby reducing Ca^2+^ overload in isolated cardiomyocytes. The present research provides comprehensive experimental evidence for the pharmacological effects of Nar, which offers a new perspective on Nar in the treatment of ischemic heart disease.

## Data Availability

The raw data supporting the conclusions of this article will be made available by the authors, without undue reservation.
